# Validation of effect of composite additive on optimized combustion characteristics of CI engine using AHP and GRA method

**DOI:** 10.1016/j.heliyon.2024.e34216

**Published:** 2024-07-06

**Authors:** Amit R. Patil, S.A. Patil, Rupali Patil, A.M. Pawar, V.N. Chougule, Kareem AboRas

**Affiliations:** a Department of Mechanical Engineering, M. E. S. College of Engineering, Savitribai Phule Pune University, Pune, MS, India; b Mechanical Engineering Department, PDEA's College of Engineering, Manjari, Bk, Pune, India; c Department of Mechanical Engineering, Pimpri Chinchwad College Of Engineering & Research, Savitribai Phule Pune University, Pune, MS, India; d Bharati Vidyapeeth Women's College of Engineering, Maharashtra, Savitribai Phule Pune University, India; e Faculty of Engineering, Alexandria University, Egypt

**Keywords:** Diesel engine, Fuel additives, Combustion, MCDM, AHP

## Abstract

The primary focus of this study is the validation of composite additives with the help of additional optimization methods and the analysis of its effect on the combustion characteristics of compression ignition (CI) engines. Previous work on the identification of the correct multi additive combination by Taguchi and the TOPSIS optimization method has shown substantial improvements in the performance and emission characteristics of CI engines. The same work was extended using the GRA Optimization method with the Multi-Criteria Decision-Making (MCDM) optimization technique known as the Analytic Hierarchy Process (AHP) to validate the optimization results from the previous optimization work. Remarkably, all optimization methods yielded consistent results, pointing to the superiority of the composite additive sample ‘D8EH6E4 hence supporting the outcome of previous work. Subsequent testing and comparison of this novel composite additive with baseline diesel fuel for combustion characteristics analysis demonstrated notable improvements in combustion parameters, including a 25 % reduction in the rate of pressure rise, an 18 % decrease in net heat release rate, and a 6 % decrease in mean gas temperature.

## 1 Introduction

The extensive utilization of CI engines has made them the predominant choice in the global transportation sector. The enhanced efficiency, reliability, and fuel economy of CI engine further dominate over petrol engines. However, in response to escalating concerns regarding air pollution and stringent governmental emission norms, researchers are expediting the work in exploring advanced methods and technologies to achieve cleaner combustion in these engines. Some of them are as below.

• Change of engine geometry to adjust fuel injection process and combustion stages
• Improvement of exhaust gas treatment
• Improving fuel properties with the help of additives;

Enhancing fuel properties presents an appealing avenue for improvement, as it obviates the need for significant design alterations and offers a straightforward, cost-effective solution. Moreover, its scalability can be readily achieved through collaboration with the oil and gas industries, facilitating cost reductions and streamlined deployment through local fuel distributors. Due to this benefits, extensive work has been performed on possibility of use of fuel additive in past years as evident from literature available on different fuel additives. T. Nibin et al. [1] and Bhavin Mehata et al. [2], found from their study that the blending of an oxygenated additive such as Di-methyl carbonate (DMC) with diesel, within a range of (5–15) % v/v, leads to significant improvements. Specifically, at a 5 % v/v DMC blend, a notable reduction of 20 % in smoke emissions was observed. Furthermore, this blending ratio resulted in a substantial 52 % reduction in soot emissions and a 25 % decrease in Soluble Organic Fraction (SOF) emissions. Yanxia and Liu Yongqi [3] investigated the effects of blending Ethylene glycol monoacetate (EGM) with DMC on diesel engine performance and emission characteristics. Notably, the study observed that reductions in smoke and carbon monooxide (CO) emissions exhibited a linear relationship with the blend proportion. However, at high loads, hydro carbon (HC) emissions were found to be elevated, with no discernible impact on oxides of nitrogen (NOx) emissions. The most significant reduction in smoke emissions was achieved when using a DMC-EGM blend at 7.5 % v/v for each component. A. R. Patil et al. [4] in their work on optimization of DMC for different CI engine parameter observed that range of 5–10 % blend is most suited for emission control with compression ratio (CR) of 18, Injection Pressure of 250 bar and Injection Timing of 23^o^bTDC but highlighted the need of multi objective optimization process. Tie Li, Masaru Suzuki [5] performed study involving Ethyl tertiary-butyl ether (ETBE)-diesel blends within the range of (10–40) % v/v, it was observed that a blend comprising 30 % v/v of ETBE led to reduced smoke emissions, achieved ultra-low NOx levels (<0.5 g/kW.hr), and improved combustion efficiency. However, this blend also exhibited a high formation of acetaldehyde, which poses environmental concerns and imposes restrictions on its practical utilization. S. Senthi kumar et al. [6] investigated the utilization of Methyl tertiary-butyl ether (MTBE) at blend proportions ranging from (2–6)% v/v, and significant reductions in HC and CO emissions were observed. The study suggested that this favourable outcome could be attributed to improved air-fuel mixing and a more efficient combustion process. Additionally, notable improvements in engine performance were documented, particularly a boost in brake thermal efficiency (BTE) when a 4 % v/v MTBE blend was employed. However, it is noteworthy that this increase in BTE was accompanied by a considerable rise in NOx emissions. P Baskar [7] examined the impact of blending diphenyl ether (DPE) (10 % v/v) and diethylene glycol monomethyl ether (DiGME) (15 % v/v) with diesel fuel. The results indicated a substantial reduction in smoke emissions, with a 50 % decrease observed for the (DPE-10 % v/v, DIGME-10 % v/v) blend and an even more pronounced 60 % reduction for the (DPE-15 % v/v, DIGME-10 % v/v) blend. Additionally, the study noted a concurrent reduction in HC and CO emissions. Cihan Bayindirli et al. [8] in their study experimentally investigated the effect of graphite oxide and graphite nanoparticle additives to cottonseed oil methyle estser on fuel properties such as viscosity, lower heating-value, density and cetane-number. The test results show BTE improvement by around 7–18 % depending on type of additives used and their percentage in blend. Brake specific fuel consumption (BSFC) has been reduced by around 7 % - 17 %. Combustion properties were also significantly affected as heat release rate (HRR) increased in range of 2–5 %. These blending shown substantial improvement in emission control as CO dropped in the range of 9–15 %, and smoke reduction in the range of 8–18 %. Based on various literature referred to and experimental work pertaining to this study, it is observed that no single additive shall have the capability to improve engine characteristics without having an adverse effect on other engine combustion parameters [9]. Use of optimization has become very common in last few decades due to trade of between different performance and emission parameter of engine. Hayri yeman et al. [10] in their work had used design of experiment (DoE) based response surface method (RSM) optimization to optimize different engine parameters working with 1-heptanol/gasoline blend. Their study concluded that optimum performance and emission values were observed with 8 % of 1-heptanol blend at engine operating condition of CR of 10:1 at 6 kg of engine load. Mehmet Celik [11] conducted a study to investigate the influence of n-heptane blending ratios with cottonseed biodiesel and manganese (Mn)-*n*-heptane blending ratios with cottonseed biodiesel on the performance and emission parameters of a CI engine. The study revealed several noteworthy findings as a blending ratio of 8 % n-heptane with cottonseed biodiesel resulted in significant improvements, including a 7.52 % increase in power, a 7.84 % increase in torque, a 2.57 % improvement in BTE, along with a 29.99 % reduction in CO emissions, a 3.16 % reduction in total hydrocarbons (THC), and a 1.18 % increase in NOx formation compared to pure cottonseed biodiesel. Furthermore, when the 8 % n-heptane blend with cottonseed biodiesel was enriched with 12 ppm of organic manganese (Mn), notable improvements were observed in engine performance. This included a 7.12 % increase in power and a 5.35 % improvement in BTE. Simultaneously, emissions were reduced, with an 8.28 % drop in CO and a 5.54 % decrease in THC. However, there was a 10.67 % increase in NOx emissions. These findings underscore the potential benefits of n-heptane blending and the addition of organic Manganese (Mn) to cottonseed biodiesel in terms of enhancing engine performance and, in some cases, reducing emissions. Mina Mehregan et al. [12] in their investigation, employed a combination of the Grey Relational Analysis (GRA) method and Taguchi DoE with an L_18_ orthogonal array to systematically optimize engine performance and emission parameters. This optimization was conducted within the framework of introducing n-hexane and n-hexadecane additives into different biodiesel classes. The research outcomes highlight the superior suitability of rapeseed biodiesel when used in conjunction with hexadecane, demonstrating its capacity to yield optimal system responses. Samet Uslu et al. [13] in their study focused on examining the blending ratio of Diethyl Ether (DEE) with palm oil, as well as the impact of injection advance and engine load on the performance and emissions of a diesel engine. The effects of these design parameters were assessed using ANOVA and signal to noise (S/N) ratio plots. The results indicated that the highest S/N ratios for performance parameters were observed at lower DEE levels and advanced injection timing. Conversely, the highest S/N ratios for emission parameters were associated with higher DEE ratios and a 35-degree crank angle (CA). Samet Uslu [14] has used RSM method for experimental testing and optimization of effect of cerium dioxide (CeO_2_) nanoparticles additives on engine performance and emission characteristics. It is observed that addition of C_e_O_2_ of 100 ppm and torque of 12Nm results in 23.125 % BTE, 130.898 ppm of CO, 786.309 ppm of NOx, and 25.654 % of smoke. A. R. Patil et al. [15] in their latest work investigated the preparation of multi additives composite and identification of optimized combination of additives which has shown substantial improvement of engine emission specially NOx with least effect on engine performance parameter. Interaction and significant studies between different additives for emission parameter like NOx and smoke along with performance parameter like BSFC and BTE were performed. The review of existing literature reveals that the addition of additives to fuel has a distinctive impact on various engine parameters. While it may enhance certain engine operating parameters, it can simultaneously have adverse effects on others. Such an additive should be optimized to exert a beneficial influence on engine combustion characteristics, which ultimately determine both engine performance and emissions. Moreover, given the disparate responses of different engine parameters, there arises a requirement to strike a balance or trade-off between them. The literature survey emphasizes the importance of employing diverse optimization approaches to achieve this delicate equilibrium [9,16]. The aim of this study is centred on development of novel composite additives for optimized combustion/performance characteristics of CI engine. The inherent limitation of fuel additives such as improving one combustion characteristics but negatively impacting another necessitates the need to identify a novel composite additive specifically tailored for diesel fuel which can exhibit significant improvement of emission characteristics of CI Engine without any adverse effect on the performance of the engine [15]. Present study conducted is extension of this work where in first stage, optimization result of previous work is validated using methods like Grey Relational Analysis (GRA) method in conjunction with Multi-Criteria Decision-Making (MCDM) optimization technique, namely the Analytic Hierarchy Process (AHP) followed by comparing the effect of optimized composite additives on CI engine combustion characteristics with base line diesel fuel.

## 2 Selection of composite additive fuel samples

As highlighted in the preceding work, composite additive combination can be selected with help of optimization method and shown significant improvement in emission characteristics without adverse effect on engine performance characteristics. The present work continues with same additives Dimethyl Carbonate, 2-Ethyl Hexyl Nitrate and ethyle acetate for composition of composite additives. The use of Taguchi DoE methodology has employed to ascertain the optimal and requisite number of additive combinations from the predetermined additive range for sample preparation. For same three design factors, each with four levels (additive 1(DMC): 4–12, additive 2(2EHN): 2–8, and additive 3(Ethyl): 1–4), resulted into utilization of same L_16_ array, encompassing a total of 16 unique combinations for the composite additives, as discussed in previous work as base samples for present work and tabulated as shown in Table 1 [15].

**Table 1:** Composition of composite Additives sample using Taguchi L_16_ array [15].

Sample no.	% Composition of additives			Sample coding
	Additive 1	Additive 2	Additive 3	
1	4	2	1	D4EH2E1
2	4	4	2	D4EH4E2
3	4	6	3	D4EH6E3
4	4	8	4	D4EH8E4
5	8	2	2	D8EH2E2
6	8	4	1	D8EH4E1
7	8	6	4	D8EH6E4
8	8	8	3	D8EH8E3
9	12	2	3	D12EH2E3
10	12	4	4	D12EH4E4
11	12	6	1	D12EH6E1
12	12	8	2	D12EH8E2
13	16	2	4	D16EH2E4
14	16	4	3	D16EH4E3
15	16	6	2	D16EH6E2
16	16	8	1	D16EH8E1

**Fig. 1:**
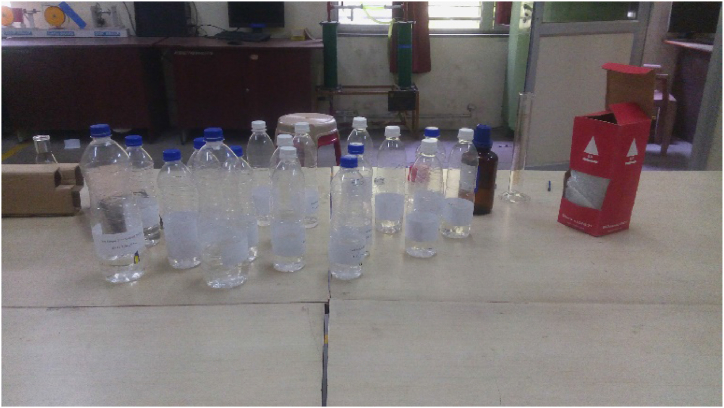
Test samples of composite additive.

Utilizing the combinations recommended by the L_16_ array, as depicted in Table 1, we proceeded to prepare sixteen test samples for experimental evaluation, as illustrated in Fig. 1. These test samples were allowed to settle for 48 h, during which no significant phase separation was observed.

## 3 Analytical chemical analysis

The introduction of fuel additives into the baseline diesel fuel inevitably leads to alterations in its physicochemical properties. Therefore, prior to begin experimental testing, it is imperative to calculate and determine the physical properties of the composite fuel that has been formulated. These properties include parameters such as density, air-fuel ratio, and heating value. Subsequently, analytical chemical analyses were carried out on these samples. This analysis has been performed in previous work using analytical method for e.g. for air to fuel Ration (A/F) calculation is done using chemical balancing and molar calculation, Heating value by energy balancing equation and density with mass balancing and tabulated [15].

## 4 Experimental investigations

**Fig. 2:**
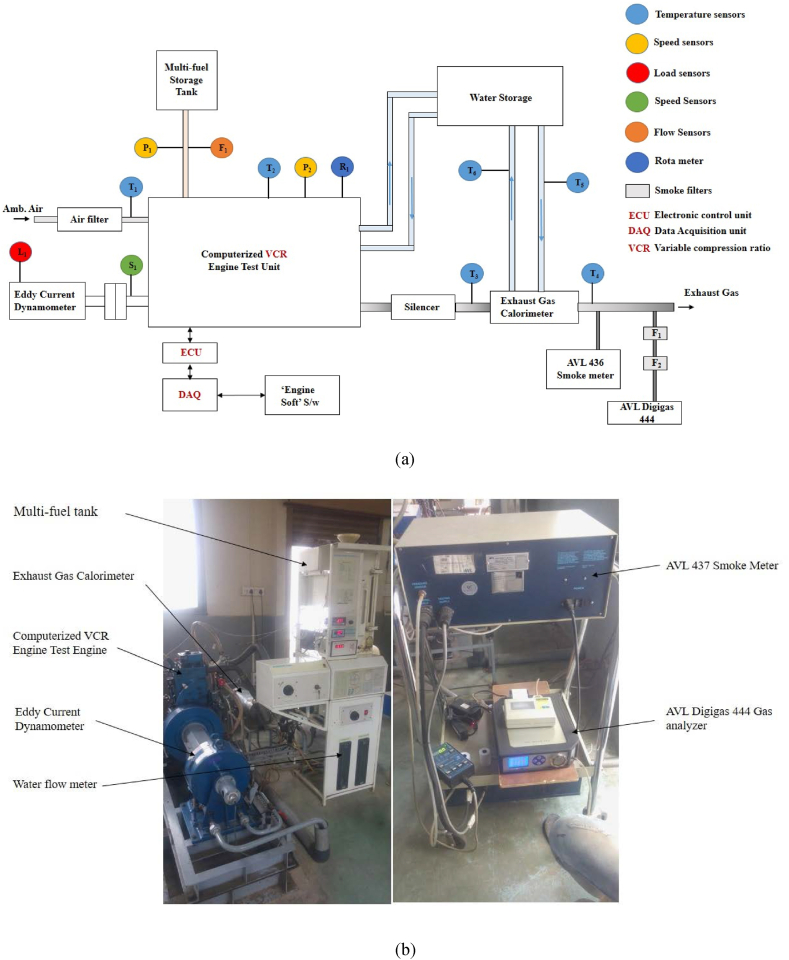
Experimental test setup (a) Schematic block diagram (b) Actual picture.

The choice of Constant Speed Variable Load Cycle is particularly well-suited for current research objectives as it was able to create different load condition during testing in previous work [15]. This choice facilitates the investigation of how different blends of composite fuel additives with baseline diesel fuel impact engine performance, both in terms of thermal characteristics and emissions, across a range of load conditions [17,18]. In present study also, the load conditions were categorized into three distinct zones, each representing varying percentages of the engine's rated power output thus simulating real life load conditions at which diesel vehicle operate especially commercial vehicles. These zones include a) Low load conditions (comprising idle and 25 % load conditions); b) Normal load conditions (encompassing 50 % and 75 % load conditions); and c) High load conditions (encompassing 100 % and 125 % load conditions) in relation to the rated power. This tripartite division is instrumental in elucidating the engine's response to the new composite additives across a spectrum of load conditions, thereby emulating real-world vehicle performance as previously suggested in the literature [19,20]. The block diagram illustrating the test setup with actual photograph is depicted in Fig. 2 (a, b). The technical details of test engine are provided in Table 2. This fully computerized test rig offers the flexibility to manipulate various operating parameters, including compression ratio (CR), fuel injection timing (FIT), fuel injection pressure (FIP), Load and exhaust gas recirculation. To monitor and document the impact of the composite additive on the combustion characteristics of CI engine during the experimental phase, a range of high-precision and calibrated sensors were employed. These sensors used for the measurements of temperature, pressure, crank angle, and more. Details regarding their respective measurement ranges and levels of precision can be found in Table 3. It is equipped to investigate the impact of a range of conventional and non-conventional fuels on engine performance. Throughout the experimentation process, individual test samples are carefully chosen and blended with diesel fuel. Subsequently, these fuel samples were then utilized for conducting combustion tests across six distinct load conditions. The trials are consistently executed at a rated speed of 1500 rpm and CR of 16. To comprehensively assess engine performance and emission characteristics at each load condition, data acquisition is facilitated through a computerized system. The experiment is replicated three times to detect and eliminate any anomalies within the test results. This procedure is subsequently reiterated for each of the sixteen composite additives sample under investigations. Also uncertainty analysis were performed for the different performance parameter and tabulated as shown in Table 4.

**Table 2:** Engine technical data

Make and model	Make Kirloskar 240,
Engine type	Single cylinder, Multi-fuel, VCR, 4 stroke, DI, water cooled
Compression ratio	12–18
Bore x stroke	87.50 mm × 110.00 mm,
Capacity	661.45 cm^3^
Rated Power	3.5 kW @ 1500 rpm,

**Table 3:** Range and accuracy levels of sensors used in Engine Test-setup.

Sr. No	Parameter	Type of Sensors	Range	Accuracy
1	Pressure	Piezo Sensors	0–350 bar	± 1 %
2	Load	Strain Gauge	0–50 kg	± 0.25 % F.S.
3	Temperature	RTD, PT 100	−50 to 400 °C	± 0.5 %
4	Temperature	Thermocouple K type	0–360 °C	± 0.5 %
5	Fuel Flow transmitter	Differential pressure Trans.	0–240 mm WC	± 0.1 %
6	Crank angle Sensor	TDC Pulse	0–360°	± 0.25 %
7	Shaft Speed	PNP Type	4.00 to 9999 RPM	± 0.05 % F.S.

**Table 4:** Accuracy and uncertainty level of test parameter

Sr. No	Test Parameter	Accuracy Range	Uncertainty (%)
1	Speed sensor	±0.25 %	
2	Load sensors	±0.25 %	
3	Fuel flow	±0.1 %	
4	Brake power		±1.56
5	Fuel consumption		±0.1
6	Break specific fuel consumption		±2.58
7	Brake thermal efficiency		±4.445

## 5 Grey relation analysis (GRA) optimization

Deng (1989) advocated the application of Grey Relational Analysis (GRA) to transform multi-response optimization problems into single-response problems. GRA is a component of the grey system theory and is particularly valuable for addressing challenges characterized by intricate interdependencies among chosen factors and variables. GRA has demonstrated its effectiveness in resolving various Multiple Attribute Decision-Making (MADM) problems by consolidating the entire spectrum of performance characteristics into a single attribute for each alternative, thereby simplifying the decision-making process into a single-attribute problem [16]. After the application of the GRA process, comparisons among alternatives with multiple attributes become more straightforward. In this study, performance parameters as BTE and BSFC were chosen for enhancement, while emission parameters such as NOx and smoke were chosen for control. Within the GRA framework, the results of performance parameters were normalized using the “higher the better” (HBT) strategy, whereas emission parameters were normalized using the “lower the better” (LTB) approach [21,22]. Subsequently, Cumulative Grey Relation Grades were computed for each test run across various load conditions and ranked as presented below in Table 5.

**Table 5:** Ranking by GRA method

Run No.	Grey relational coefficient				Grey Relation Grade	Rank
	BSFC	NOx	Smoke	BTE		
1	1.000	0.333	0.339	0.742	0.124	11
2	0.500	0.467	0.667	0.655	0.140	6
3	0.333	0.397	0.667	0.373	0.116	12
4	0.333	0.365	1.000	0.333	0.137	8
5	0.600	0.398	0.521	0.612	0.124	10
6	0.500	0.555	0.809	0.512	0.154	5
7	1.000	1.000	0.655	0.903	0.220	1
8	0.500	0.571	1.000	0.474	0.169	4
9	0.375	0.606	0.667	0.356	0.138	7
10	0.429	0.464	0.500	0.424	0.116	13
11	1.000	0.539	0.594	1.000	0.173	2
12	0.750	0.442	0.333	0.772	0.126	9
13	0.375	0.407	0.413	0.403	0.101	16
14	0.429	0.445	0.373	0.461	0.106	15
15	0.600	0.653	0.826	0.516	0.169	3
16	0.500	0.455	0.432	0.487	0.115	14

## 6 MCDM optimization method

**Fig. 3:**
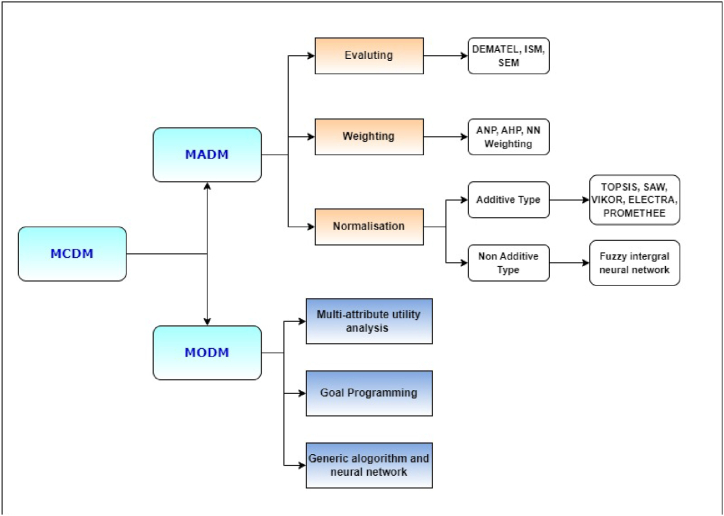
Classification of MCDM [24].

The investigation reveals that the substantial influence of various engine parameters on the performance of fuel additives across different engine output parameters necessitates a meticulous and comprehensive selection and analysis process when considering their use with diesel. This scenario characterizes the present study as a multi-attribute problem, demanding optimization through Multi-Criteria Decision-Making (MCDM) techniques. Multi-attribute optimization methodologies have gained prominence due to their proven effectiveness in numerous engineering and scientific domains, where achieving an optimal solution requires a delicate balance between conflicting outcomes. This complexity arises from the need to minimize certain output parameters while simultaneously maximizing others, creating intricate trade-off relationships between them [23]. To solve and optimize the output in such situation, different types of MCDM methods are present to choose and apply as shown in Fig. 3.

Multi-Attribute Decision-Making (MADM) optimization endeavours to select the most suitable alternative from a pool of multiple attributes that often compete with one another. Various procedures are available for this purpose, as depicted in Fig. 3. In this study, we have opted for the Analytic Hierarchy Process (AHP) as the chosen method for optimization.

## 7 Analytical hierarchy approach (AHP) optimization

**Fig. 4:**
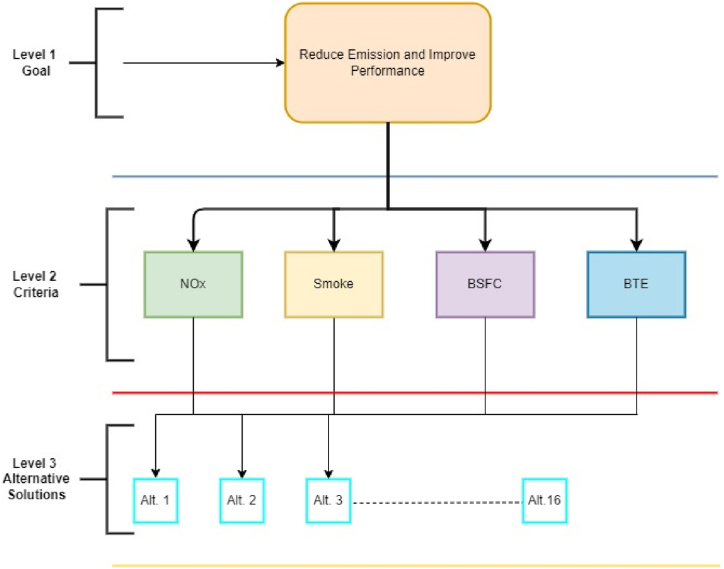
Hierarchical decomposition process.

As highlighted in the literature review, previous research has extensively employed the Analytic Hierarchy Process (AHP) for optimizing multi-attribute problems (MADM). In present study, the optimization process was streamlined by leveraging graph theory to establish digraph relationships using the performance and emission attribute studied previously [15]. A notable advantage of this approach is its ability to assign priorities to attributes relative to others based on both past experience and human judgment, which can be substantiated through consistency analysis. These validated attribute weights are subsequently utilized to construct a judgment matrix, which is then solved to determine rankings that facilitate the identification of the optimized combination of design factors. The significance of this hierarchical decomposition is illustrated in Fig. 4 enhancing our comprehension of the problem and simplifying the decision-making process, thereby necessitating verification of the chosen criteria and alternatives [[25], [26], [27], [28]].

The permanent function for decomposition shown in Fig. 4 is then converted in the form of permanent matrix as represented by Equation (1).

(1) $$\left[P\right]=\left[\begin{array}{cccc} D_{1} & a_{12} & a_{13} & a_{14}\\ a_{21} & D_{2} & a_{23} & a_{23}\\ a_{31} & a_{32} & D_{3} & a_{32}\\ a_{41} & a_{42} & a_{43} & D_{4} \end{array}\right]$$

The permanent function matrix plays a crucial role in facilitating the precise allocation of various design factors and the assignment of attribute weights based on their relevance to the problem at hand. Notably, the presence of exclusively positive signs in the function matrix, in contrast to determinants, ensures that no crucial information is omitted or overlooked [29]. The Performance Index (PI) is an indication of consistency by which an optimum design parameters combination can be identified for a given problem case studies [23,30]. The significance and weighting of individual criteria can vary depending on the specific circumstances. In this study, the initial step involves conducting pairwise comparisons to determine the relative importance of each criteria based on the research objectives. These relative priorities were assigned using a numerical value called Saaty's Scale as shown in Table 6.

**Table 6:** Saaty's pairwise comparison scale [31]

Verbal Judgment	Numeric Value	
	$a_{ij}$	$a_{ji}$
Highly important	9	1/9
	8	1/8
Very strongly more important	7	1/7
	6	1/6
Strongly more important	5	1/5
	4	¼
Moderately more important	3	1/3
	2	½
Equal importance	1	1

In the present work, the primary objective is to reduce NOx emissions and smoke from CI engines without adversely affecting its performance characteristics. Consequently, the control of NOx emissions takes precedence over smoke control, and it has been assigned a value of ‘2′ to account for the trade-off between the two objectives. Simultaneously, the importance of reducing NOx emissions surpasses that of engine parameters like BSFC and BTE, and it has been designated a value of ‘3′ relative to them. In the context of assigning numerical values to the significance of smoke reduction concerning other response parameters, smoke reduction is somewhat less critical than NOx reduction but more crucial than optimizing BTE and BSFC. Hence, it has been allotted a value of '½' concerning NOx, ‘2′ concerning BSFC, and BTE. Same follow for other parameters which are shown in Table 7.

**Table 7:** Pairwise comparison matrix of criteria for present problem situation [16,28].

	NOx	Smoke	BTE	BSFC
NOx	1	2	3	3
Smoke	½	1	2	2
BTE	1/3	½	1	½
BSFC	1/3	½	2	1

Next assigned priorities are converted into normalized matrix from original judgment as shown in Table 8.

**Table 8:** Criteria judgment matrix

	NOx	Smoke	BTE	BSFC
NOx	1.000	2.000	3.000	3.000
Smoke	0.500	1.000	2.000	2.000
BSFC	0.333	0.500	2.000	1.000
BTE	0.333	0.500	1.000	0.500

Each criteria value is normalized using Equation (2).

(2) $$X_{ij}=\frac{x_{ij}}{\sum_{1}^{j}x_{ij}}$$

Where.

X_ij_ = Normalized criteria of i ^th^ column of jth row.

These values are replaced with criteria weight as shown in Table 9.

**Table 9:** Normalized matrix

	NOx	Smoke	BTE	BSFC	Local Priority
NOx	0.462	0.500	0.375	0.462	0.450
Smoke	0.231	0.250	0.250	0.308	0.260
BSFC	0.154	0.125	0.250	0.154	0.171
BTE	0.154	0.125	0.125	0.077	0.120

Average/Overall Priority is identified for individual criteria in this problem by taking average of row value for each criteria as shown in Table 9. It indicates that NOx holds the highest importance among the criteria, accounting for 45 % of the overall significance, followed by smoke and BSFC. These prioritizations reflect our subjective judgments and preferences. However, it's worth noting that since these assigned values are influenced by individual inclinations, a degree of inconsistency in the final judgment matrix is inevitable. This allowable inconsistency is quantified by the Consistency Ratio (CRa). The Analytic Hierarchy Process (AHP) aids in the computation of the Consistency Ratio (CRa) through the utilization of the Consistency Index (CI) derived from our decision matrix and the Consistency Index of a randomly generated matrix (RI). The RI represents the average CI derived from 500 randomly populated matrices. Saaty's (2012) offers the calculated RI values for matrices of different sizes as shown in Table 10 [23,25].

**Table 10:** Consistency indices table for RI

N	3	4	5	6
RI	0.580	0.900	1.120	1.240

Following the assignment of importance to all the design parameters in the problem, the subsequent step involves allocating comparative priorities (weights) to these design parameters. This allocation is based on both past experience and the perceived importance or significance of different design factors, according to the objectives of the present study. It is relative values as the obtained priorities are measured with respect to each other. The result is shown in Table 11.

**Table 11:** Priority as criteria weight

Criteria Weight	NOx	Smoke	BTE	BSFC
	0.450	0.260	0.120	0.171
NOx	1.000	2.000	3.000	3.000
Smoke	0.500	1.000	2.000	2.000
BSFC	0.333	0.500	2.000	1.000
BTE	0.333	0.500	1.000	0.500

The priority will become criteria weight and each criteria value is multiplied by weight to get weight sum or overall priority as shown in Table 12.

**Table 12:** Calculation of weighted column & sum

	NOx	Smoke	BTE	BSFC	Overall Priority
NOx	0.450	0.519	0.361	0.512	1.841
Smoke	0.225	0.260	0.240	0.341	1.066
BSFC	0.150	0.130	0.240	0.171	0.691
BTE	0.150	0.130	0.120	0.085	0.485

Next maximum Eigen (*λ*_max_) value is identified by taking the ratio of weighted sum by prioritizing each design criteria and then averaging these values. Dividing the weighted sum of factor by respective priority will give *λ*_max_ which are tabulated as shown in Table 13.

**Table 13:** Calculation of maximum Eigen value

Criteria	Weighted sum	Priority	Λ_max_
NOx	1.8413	0.4495	4.096257
Smoke	1.0661	0.2596	4.106481
BSFC	0.6907	0.1707	4.046948
BTE	0.4852	0.1202	4.036667
Total			16.28635
*λ*_max_			4.071588

The *λ*_max_ is then used to calculate Consistency index (CI) by using Equation 3.

(3) $$CI=\frac{\lambda_{\max}-n}{n-1}$$

CI=0.024,

Where, n = number of compared element (since factors are four hence n = 4)

(4) $$\mathrm{CR}=\left(\frac{\text{CI}}{\text{RI}}\right)$$

$$CR=\frac{0.024}{0.9}=0.027$$

As per Table 10 for present situation of 4 by 4 matrix, we take value of RI as 0.9 [32] in Equation (4). In the current context, where the inconsistency value (CR) stands at 0.027, which is below the threshold of 0.10, we can confidently affirm that our judgment matrix exhibits rational consistency. Consequently, we can proceed with the decision-making process employing the AHP technique [16]. So acceptable judgment matrix component for further decision making and optimization process is shown in Table 14.

**Table 14:** Final attribute matrix for decision making

	NOx	Smoke	BTE	BSFC
NOx	D1	2.000	3.000	3.000
Smoke	0.500	D2	2.000	2.000
BSFC	0.333	0.500	2.000	D3
BTE	0.333	0.500	D4	0.500

This allocation was transformed into a performance matrix, which quantifies the degree of simplicity in selecting an optimal combination of performance parameters for a given system or process. In our current research study, the acceptable Performance Index (PI) is expressed by Equation (5). The diagonal components (D_1_, D_2_, D_3_, and D_4_) in Equation (5) are determined by substituting them with the normalized attribute values for each test run. Subsequently, these performance matrices are solved through complex and time-consuming mathematical calculations to derive the performance index (PI) value. [33] [34] [23].

(5) $$\left[P\right]=\left[\begin{array}{cccc} D_{1} & 2 & 3 & 3\\ 0.55 & D_{2} & 2 & 2\\ 0.33 & 0.5 & D_{3} & 2\\ 0.33 & 0.5 & 0.5 & D_{4} \end{array}\right]$$

The performance index (PI) is a measure of the easiness with which an optimum combination of operative parameters can be chosen for a given system or process. Table 15 shows sample permanent matrix for run No. 1 & 4 for idle load condition with their calculated PI values.

**Table 15:** Sample PI matrix tables with PI values

Run no 1					Run no 4				
	NOx	Smoke	BSFC	BTE		NOx	Smoke	BSFC	BTE
NOx	0.715	2.000	3.000	3.000	NOx	0.494	2.000	3.000	3.000
Smoke	0.500	0.152	2.000	2.000	Smoke	0.500	0.455	2.000	2.000
BSFC	0.333	0.500	0.122	2.000	BSFC	0.333	0.500	0.643	2.000
BTE	0.333	0.500	0.500	0.072	BTE	0.333	0.500	0.500	0.554
PI	13.0524				PI	16.861			

Solving the permanent matrix yields a performance index value. The cumulative performance index values for the 16 test samples under various load conditions were calculated and presented in Table 16. These values were then ranked based on the magnitude of the performance index, with higher values receiving a higher rank, indicating the most effective composite additive for optimizing conditions. In this context, run number ‘7′ featuring the composite sample D8EH6E4 achieved the highest performance index at 23.2306, earning it the top rank of ‘1.’ This suggests that D8EH6E4 is the most suitable composition for optimizing the operation of a CI engine, as it maximizes NOx reduction and minimizes smoke emissions without adverse effects.

**Table 16:** Ranking based on PI value

Run no.	PI	Rank
1	13.0524	14
2	17.4589	7
3	12.7426	15
4	16.861	10
5	18.8784	4
6	17.4589	7
7	23.2306	1
8	15.6458	11
9	14.4024	13
10	11.4751	16
11	15.2007	12
12	17.4703	6
13	17.4105	9
14	18.232	5
15	20.7539	3
16	21.5154	2

## 8 Comparison of optimization results

When result of both optimization method is compared as shown in Table 17, it is observed that test run using sample number ‘7’ is having the first rank as per GRA method and also confirmed by AHP Ranking method thus validating the result of best possible solution for the previous optimization work.

**Table 17:** Ranking based on PI value

Run no.	GRA rank	AHP rank
1	11	14
2	6	7
3	12	15
4	8	10
5	10	4
6	5	7
7	1	1
8	4	11
9	7	13
10	13	16
11	2	12
12	9	6
13	16	9
14	15	5
15	3	3
16	14	2

## 9 Effect of composite additive on combustion characteristics

The performance and emissions of an engine hinge primarily on the combustion process transpiring within the engine cylinder, which in turn influences various combustion process characteristics. To capture these intricacies, a suite of high-precision sensors including pressure, temperature, and position sensors were meticulously installed. Utilizing a Data Acquisition (DAQ) system, data pertaining to combustion temperature and pressure were recorded at distinct crank positions for ten power cycles. This process was replicated for both baseline diesel and diesel containing the proposed composite additive. The same procedure was further iterated for different test load conditions. Subsequently, these recorded data were employed to generate a series of graphs depicting Maximum Combustion Peak Pressure, Maximum Rate of Pressure Rise, Maximum Net Heat Release, and Mean Gas Temperature.

### 9.1 Combustion pressure vs. crank angle

**Fig. 5:**
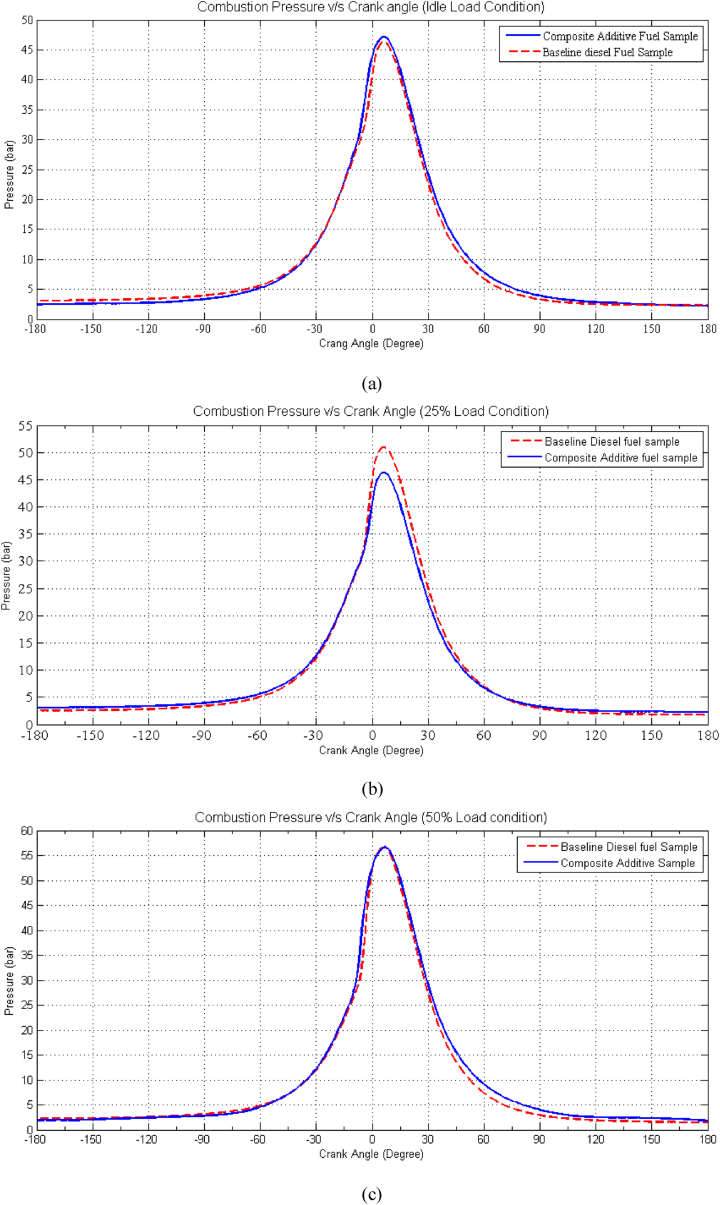

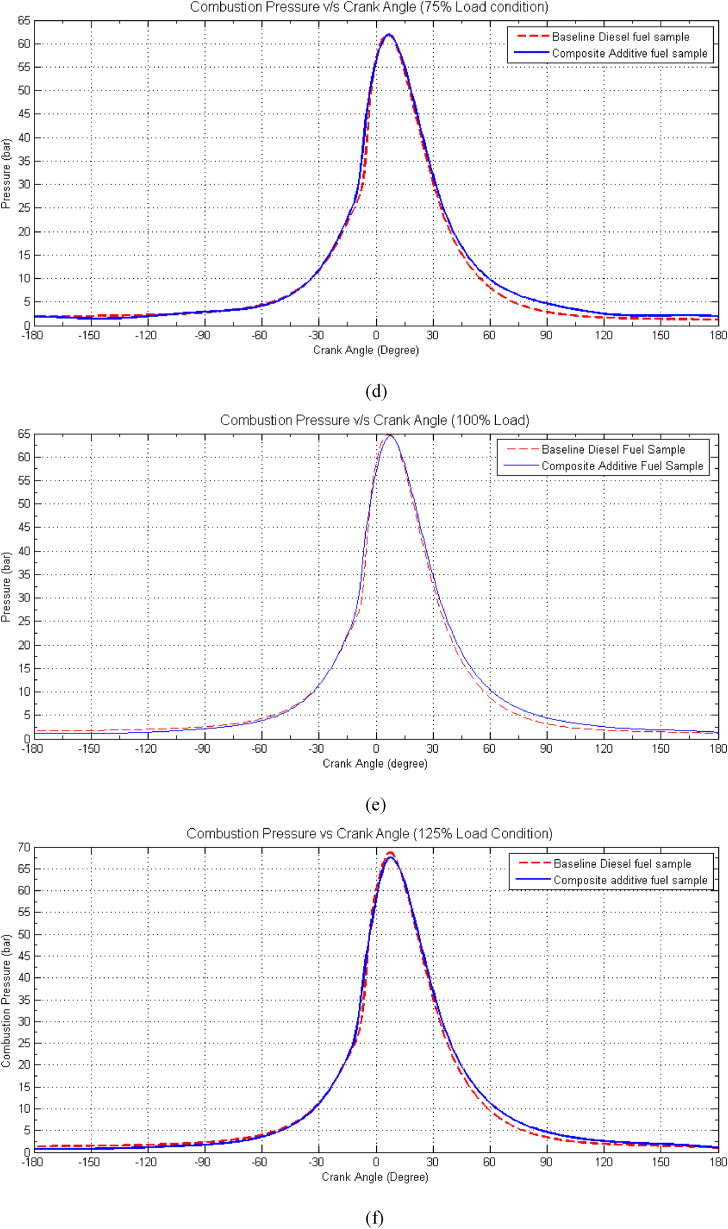
Effect of Composite Additive on Combustion Pressure vs. Crank Angle for different load conditions. (a) Idle Load condition, (b) 25 % Load Condition, (c) 50 % Load Condition, (d) 75 % Load condition, (e) 100 % Load Condition and (f) 125 % Load Condition.

As seen from Fig. 5(a-f)**,** the combustion pressure observed in the composite additive fuel samples consistently registers lower values than that of the baseline diesel fuel across all tested load conditions. This phenomenon can be attributed to the lower heat capacity of the composite additive fuel, stemming from its reduced calorific heating value (CV) and the leaner mixture it creates. For instance, at a low load condition of 25 %, the peak pressure for baseline fuel reaches 52 bar, while for the composite additive, it measures 47 bar. However, at this load condition, both baseline diesel and composite additive fuel samples ultimately achieve the same peak pressure. This results in a reduced availability of heat energy for conversion into pressure energy. The inclusion of 2-EHN in the composite additive improves the ignition quality of the fuel sample, thereby mitigating the decline in combustion pressure compared to that of the baseline diesel fuel. This suggests that the power rating of the engine utilizing the proposed composite fuel sample closely approximates that of a conventional engine using baseline diesel fuel. Additionally, the combustion stages observed with the proposed composite additive align closely with those of a conventional diesel engine, with peak pressure occurring at approximately 50° after Top Dead Center (aTDC).

### 9.2 Rate of pressure rise v/s crank angle

**Fig. 6:**
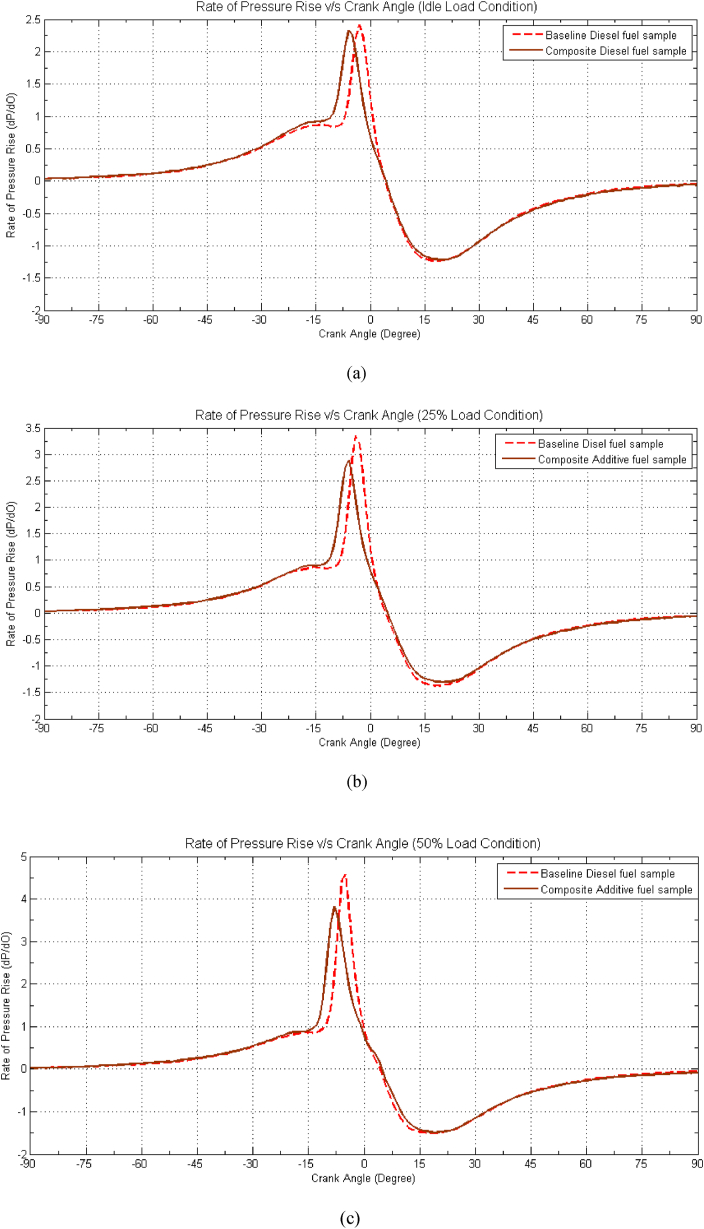

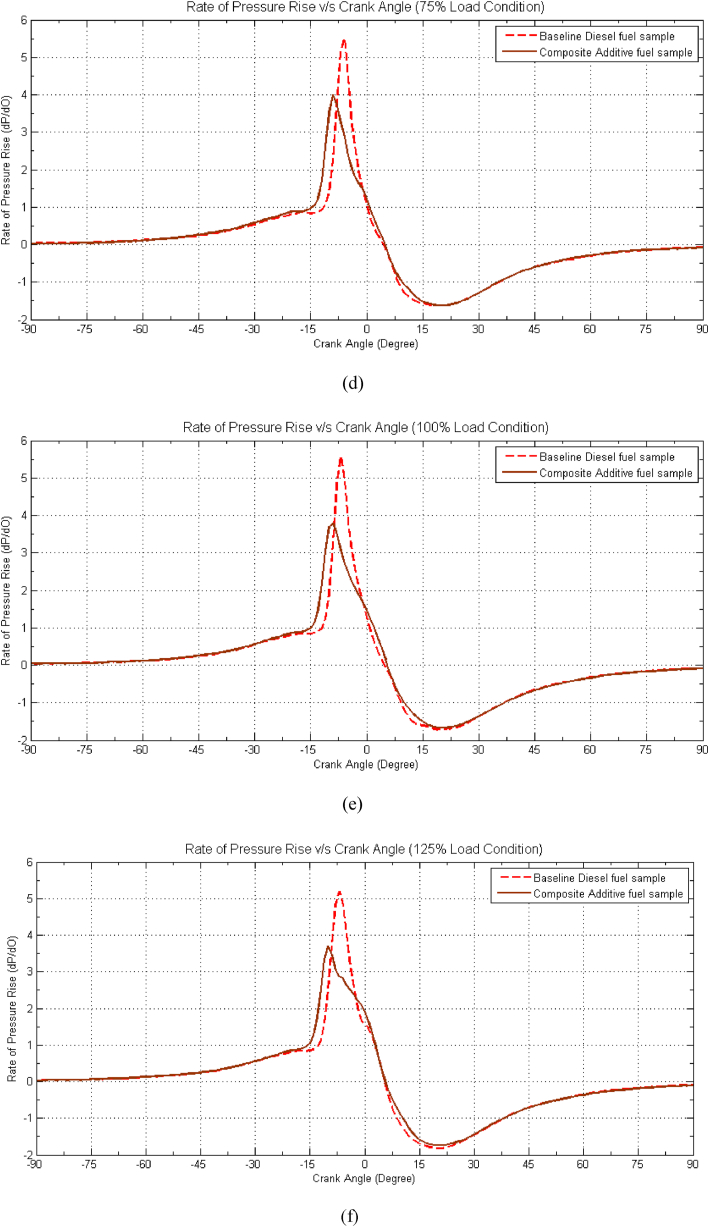
Effect of Composite Additive on Rate of Pressure Rise vs. Crank angle for different load conditions. (a) Idle Load condition, (b) 25 % Load Condition, (c) 50 % Load Condition, (d) 75 % Load condition, (e) 100 % Load Condition and (f) 125 % Load Condition.

Fig. 6(a–f) provides valuable insights into the behaviour of the composite fuel sample in comparison to baseline diesel fuel. Notably, the composite fuel sample exhibits a reduced pressure rise rate across various load conditions, with a decrease of approximately 0.25 bar/degree for idle load, 0.5 bar/degree for 25 % load, 0.58 bar/degree for 50 % load, 0.6 bar/degree for 75 % load, 0.62 bar/degree for 100 % load, and a more substantial drop of 0.7 bar/degree during overload conditions when contrasted with baseline diesel fuel. Examining Fig. 6, it becomes evident that the Maximum Rate of Pressure Rise (RPR) for the optimized composite sample is displaced by approximately −2 to −3° before Top Dead Center (bTDC) relative to baseline diesel fuel. This shift in the RPR is primarily attributed to the lower heating value of the composite sample, which contributes to a decline in the maximum rate of pressure rise during the combustion process. However, it's noteworthy that there is an improvement in the pressure rise rate during the initial stage of combustion, attributable to the enhanced ignition quality of the fuel, facilitated by the presence of DMC and 2-EHN as catalytic effect of additive improve the reaction of fuel with more oxygen [11]. [35] The drop in RPR is most pronounced in higher load conditions, reaching up to 20 %. Furthermore, the peak of pressure rise rate shifts away from Top Dead Center (TDC), moving into the compression stroke. This shift is a consequence of the relatively delayed combustion process and heat release, occurring predominantly in the mixing-controlled phase of combustion at higher engine load conditions. Additionally, with increasing engine load, a rise in in-cylinder temperature is observed, likely reducing ignition delay and resulting in earlier ignition of the premixed charge. This, in turn, contributes to a reduction in pressure rise rate.

### 9.3 Neat Heat release (NHR) vs. crank angle

In an Internal Combustion Engine (ICE), the combustion process can be delineated into three distinct phases. The initial stage, characterized by a rapid combustion rate, is relatively short-lived in terms of crank angle. The second phase, known as the primary High-Rate-of-Rise (HRR) period, concludes within the range of 40–50° crank angle (CA). It is worth noting that the first and second phases collectively account for approximately 80 % of the total fuel energy release. Finally, the third phase of combustion is responsible for releasing the remaining 20 % of the total fuel energy [8]. [36]

**Fig. 7:**
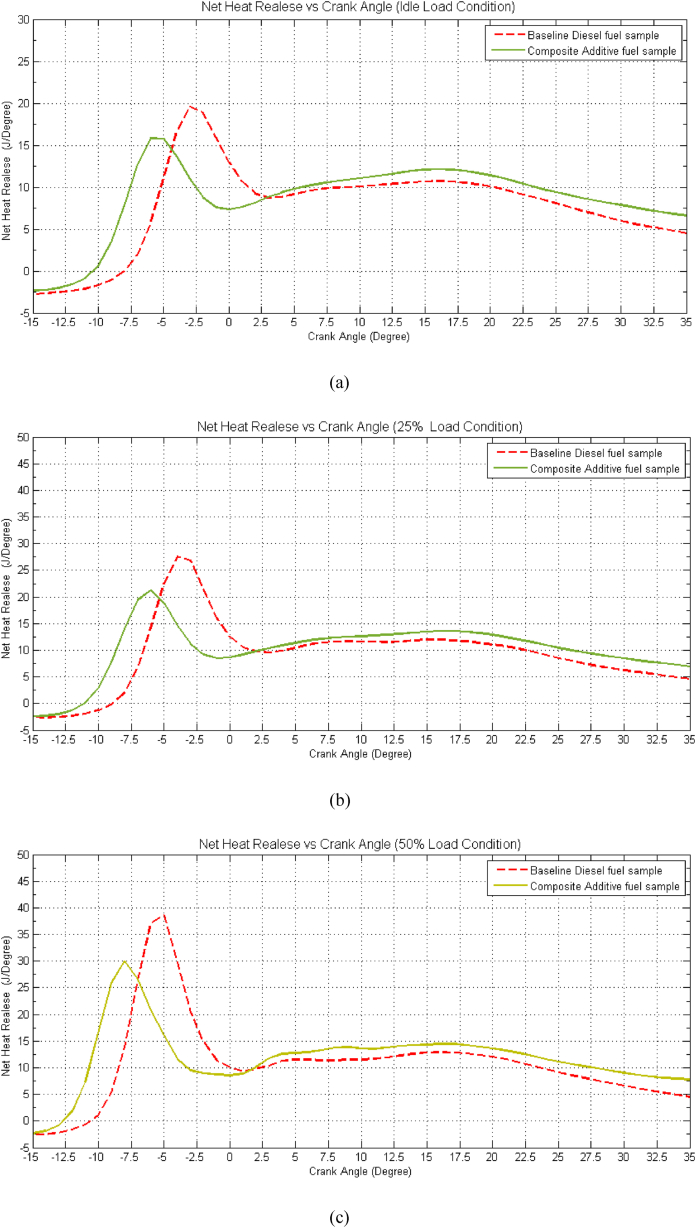

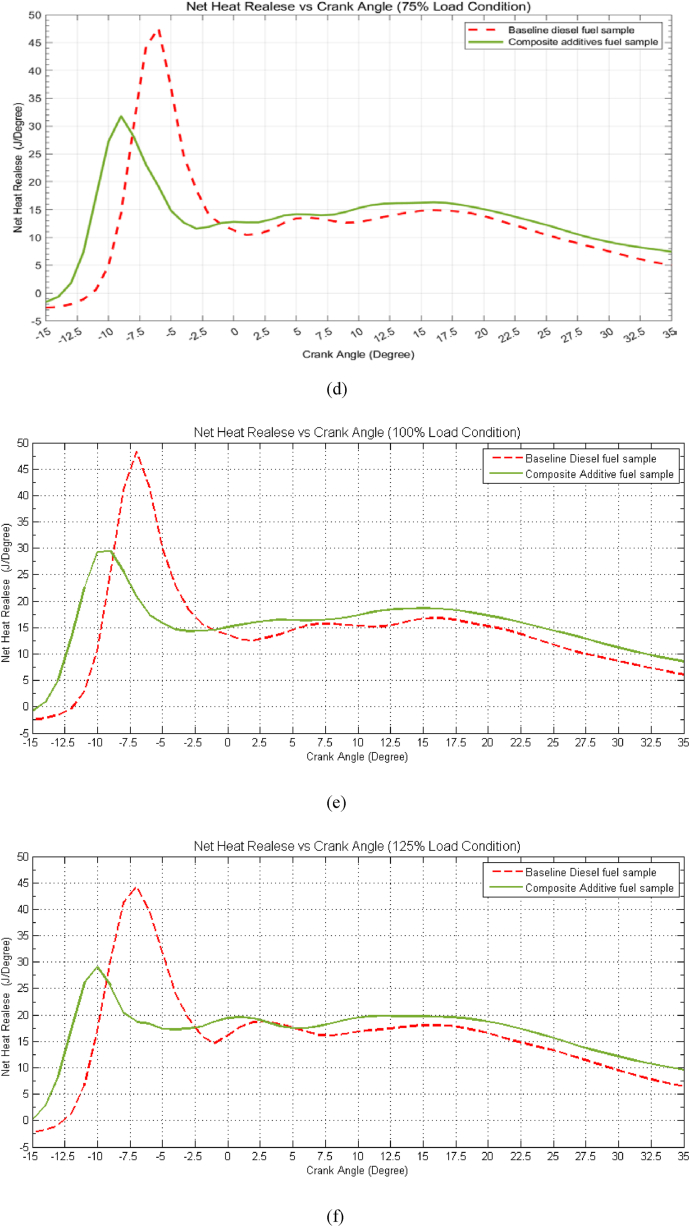
Effect of composite additive on Neat Heat release (NHR) vs. crank angle for different load conditions. (a) Idle Load condition, (b) 25 % Load Condition, (c) 50 % Load Condition, (d) 75 % Load condition, (e) 100 % Load Condition and (f) 125 % Load Condition.

In Fig. 7(a–f), the variation in net heat release (NHR) versus crank angle (CA) is depicted across different load conditions for both the composite fuel sample and the baseline diesel fuel. It is noteworthy that the rate of heat release is comparatively lower during low-load conditions and escalates as the load increases. This phenomenon is attributed to the enrichment of the air-fuel mixture, observed in both test samples. Moreover, a discernible reduction in heat release is observed in the composite fuel compared to baseline diesel fuel, which can be ascribed to the presence of additives with a lower calorific value. This discrepancy in heat release is less pronounced at idle and low load conditions, up to 50 %, where it hovers around 18 %. However, it becomes considerably more significant at higher load conditions, reaching approximately 30 % [37]. The calorific value of the optimized composite fuel sample is nearly 8 % lower than that of the baseline fuel, signifying a lower rate of heat energy release during the combustion process. Consequently, at higher loads, due to the enrichment of the air-fuel mixture, the calorific value of the charge in the combustion chamber is substantially diminished compared to that of the baseline diesel.

### 9.4 Mass fraction burn vs. crank angle

**Fig. 8:**
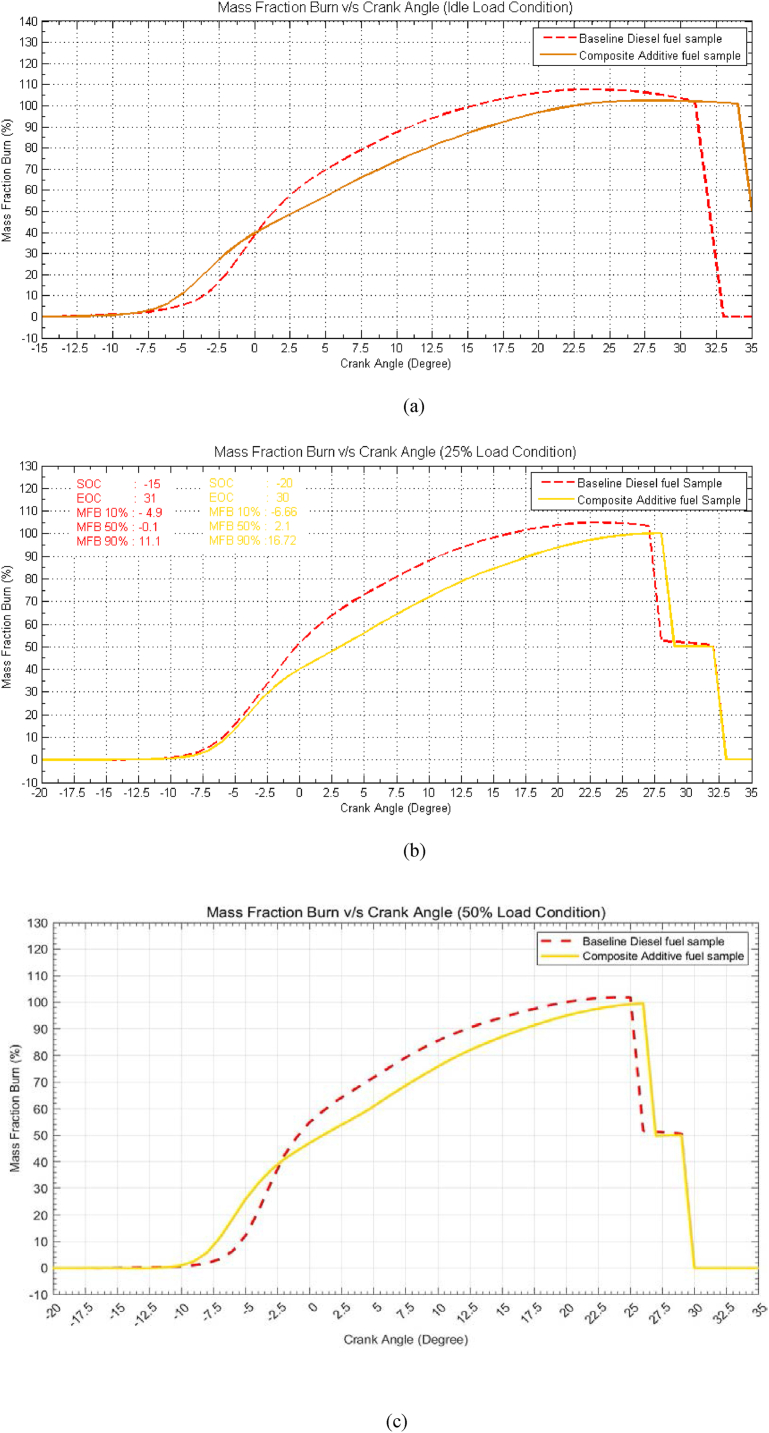

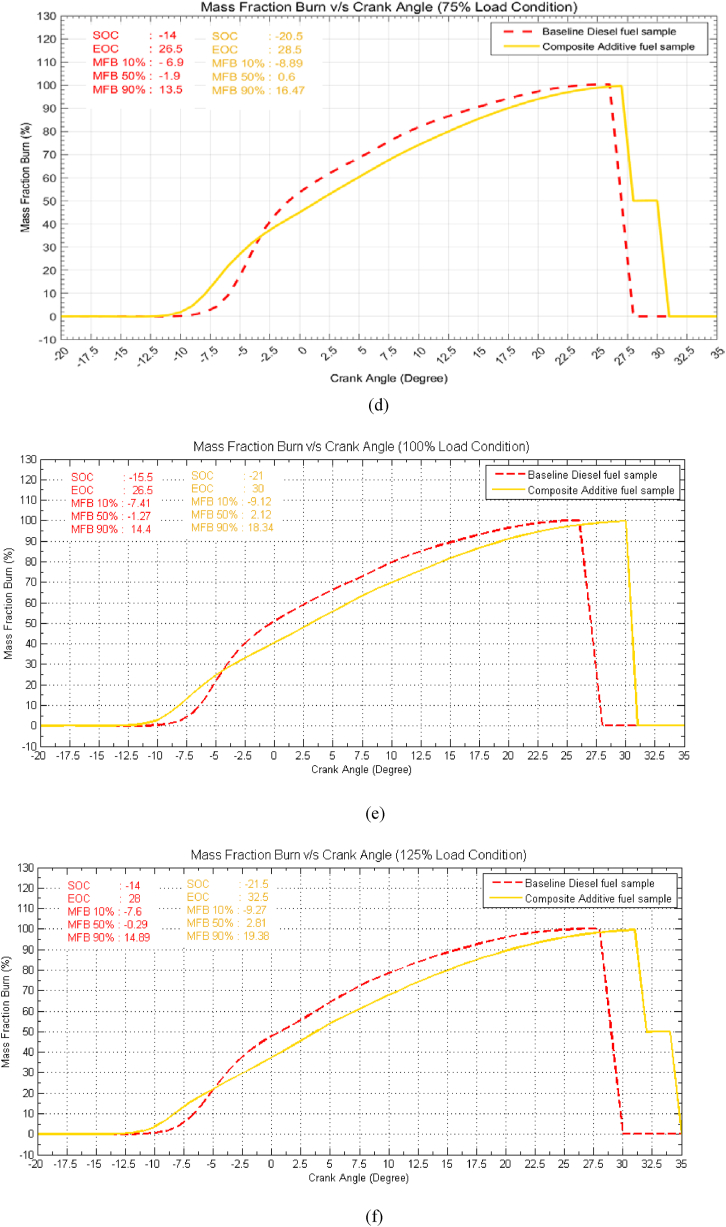
Effect of composite additive mass fraction burn vs. crank angle for different load conditions. (a) Idle Load condition, (b) 25 % Load Condition, (c) 50 % Load Condition, (d) 75 % Load condition, (e) 100 % Load Condition and (f) 125 % Load Condition.

Fig. 8(a–f) comprises multiple graphs depicting the mass fraction burn (MFB) rate at different load conditions for three distinct stages of burning: the combustion of 10 % of the fuel mass, 50 % of the fuel mass, and finally, 90 % of the fuel mass. The time required to reach these stages is presented in terms of crank angle for both baseline diesel (indicated by dashed lines) and the composite additive fuel sample (indicated by solid lines). Upon examination of Fig. 8, it is evident that at idle load conditions, the composite additive fuel sample exhibits unique behaviour. Specifically, the 10 % mass fraction burn occurs at −6.26° bTDC, which is approximately 1.7° earlier than baseline diesel. Conversely, the 50 % mass fraction burn occurs at 1.91° aTDC, representing a delay of approximately 1.5°, while the 90 % mass fraction burn transpires at 15.39° aTDC, signifying a delay of about 5.4° when compared to baseline diesel fuel. This trend persists across varying load conditions. Due to the presence of the composite additive, the delay in the 50 % mass fraction burn extends from around 1.5 to 2° at low load conditions and increases to 3.1° under overload conditions. Similarly, the 90 % mass fraction burn process experiences a delay of approximately 5.4 to 4.5° at low load conditions but decreases to around 4.4° at overload conditions. The graphs also reveal that, for the composite additive fuel sample, the commencement of combustion advances by approximately 2 degrees of crank angle at low load conditions, which escalates to approximately 5.5–6.5 degrees of crank angle at higher load conditions when compared to baseline diesel. The conclusion is that the combustion process concludes later by approximately 4° at low load conditions and about 2° at 75 % load, which increases to 4.5° for overload conditions. For instance, at a load condition of 75 %, the combustion process for the composite additive fuel sample initiates at −17.5° bTDC, which is advanced by 4°, while the conclusion of the combustion process occurs at 28.5° aTDC, indicating a delay of approximately 2° compared to baseline diesel fuel. This trend signifies that the use of the composite additive extends the duration of the combustion process, resulting in increased time for fuel-air interaction. Consequently, this promotes a more homogeneous mixture and enhances combustion. Notably, the burning rate is observed to decrease due to the addition of composite additives, which can be attributed to the lower calorific value of the composite additive fuel sample. This reduced burning rate between 10 % MFB and 50 % MFB contributes to a decrease in combustion temperature, as it allows for increased contact between fuel and air, enhancing charge cooling and reducing the likelihood of spontaneous combustion. Lower combustion temperatures are responsible for reduced NOx emissions, further contributing to lower emissions and improved engine performance.

## 10 Conclusion

The findings of the current study can be summarized as follows.

• The integration of the Grey Relational Analysis (GRA) method and the Analytic Hierarchy Process (AHP) method in the Multi-Criteria Decision Making (MCDM) optimization, produced result which indicate that sample D8EH6E4 is optimized combination thus validating the optimization result of previous work.
• Examination of combustion data revealed that the Maximum Net Heat Release (NHRMax) for the proposed composite additive decreased by 23 % across most load conditions due to the lower heating value of the composite additive compared to baseline diesel fuel.
• The reduction in NHRMax led to a slight decrease in Peak Combustion Pressure (CPMax) and a 6 % reduction in Mean Gas Temperature (MGT) compared to baseline diesel fuel. Additionally, the optimized sample demonstrated improvements in latent heat of vaporization and affected the local equivalence ratio in the combustion zone, thereby reducing local hotspots.
• The composite additive resulted in a 25 % reduction in Rate of Pressure Rise (RPR) at higher load conditions, and the decreased NHR contributed to cooling the combustion process, leading to a reduction in combustion temperature.
• The current study is confined to engines without Exhaust Gas Recirculation (EGR) capability. However, it can be extended to investigate the influence of EGR on the behaviour of the composite additive concerning engine combustion, performance, and emission characteristics.

In summary, our present research has successfully validated the optimization result of previous work, confirming the suitability of composite additive D8EH6E4, in demonstrating a distinct capability to impact combustion characteristics across diverse load conditions. Through collaboration between oil industries and local fuel dealers, this novel additive can be made readily accessible to diesel vehicles at a cost-effective rate, thus contributing to improved engine combustion and the mitigation of emission gases. The current study is conducted under predefined engine operating parameters such as FIT, FIP, and CR. Future research could extend the investigation to explore the effects under varying engine operating parameters, necessitating the identification of optimized test parameters.

## Availability of data and material

Data will be made available on reasonable request.

## Research funding

This research work did not receive any funding for carrying out the experimental work.

## CRediT authorship contribution statement

**Amit R. Patil:** Writing – review & editing, Writing – original draft, Validation, Methodology, Investigation, Data curation, Conceptualization. **S.A. Patil:** Writing – review & editing, Visualization, Formal analysis, Data curation. **Rupali Patil:** Writing – review & editing, Visualization, Formal analysis, Data curation. **A.M. Pawar:** Validation, Supervision, Formal analysis. **V.N. Chougule:** Writing – review & editing, Supervision, Formal analysis. **Kareem AboRas:** Writing – review & editing, Supervision.

## Declaration of competing interest

The authors declare that they have no known competing financial interests or personal relationships that could have appeared to influence the work reported in this paper.
